# Essential Oil and Juice from Bergamot and Sweet Orange Improve Acne Vulgaris Caused by Excessive Androgen Secretion

**DOI:** 10.1155/2020/8868107

**Published:** 2020-10-06

**Authors:** Peng Sun, Liang Zhao, Nanhai Zhang, Chengtao Wang, Wei Wu, Arshad Mehmood, Liebing Zhang, Baoping Ji, Feng Zhou

**Affiliations:** ^1^Beijing Key Laboratory of Functional Food from Plant Resources, College of Food Science and Nutritional Engineering, China Agricultural University, Beijing 100083, China; ^2^Beijing Advance Innovation Center for Food Nutrition and Human Health, Beijing Engineering and Technology Research Center of Food Additives, Beijing Technology and Business University, Beijing 100048, China; ^3^College of Engineering, China Agricultural University, Beijing 100083, China

## Abstract

Acne vulgaris is one of the most common chronic inflammatory skin diseases. Bergamot and sweet orange are rich in nutritional and functional components, which exhibit antioxidant, anti-inflammatory, and antiapoptotic effect. The aim of this study was to evaluate the potential effect of bergamot and sweet orange (juice and essential oil) on acne vulgaris caused by excessive secretion of androgen. Eighty male golden hamsters were randomly divided into 10 groups and received low or high dose of bergamot and sweet orange juice and essential oil, physiological saline, and positive drugs for four weeks, respectively. Results showed that all interventions could improve acne vulgaris by reducing the growth rate of sebaceous gland spots, inhibiting TG accumulation, decreasing the release of inflammatory cytokines (notably reducing IL-1*α* levels), promoting apoptosis in the sebaceous gland, and decreasing the ratio of T/E_2_. Among them, bergamot and orange essential oil may have better effects (dose dependent) on alleviating acne vulgaris than the corresponding juice. In view of the large population of acne patients and the widespread use of sweet orange and bergamot, this study is likely to exert an extensive and far-reaching influence.

## 1. Introduction

Acne vulgaris is a prevalent dermatologic disease, mainly distributed in the pilosebaceous units including the face, neck, chest, back, and shoulders. The clinical manifestations of acne vulgaris are mainly seborrheic, noninflammatory skin lesions, inflammatory lesions, and varying degrees of scarring [1, 2]. More than 85% of young individuals are affected by acne vulgaris worldwide and can suffer from the disease into adulthood [3]. Although acne vulgaris is not life-threatening, this disease can have a huge impact on patients' psychosocial and physical health. Acne vulgaris results from the androgen-induced sebum production, altered keratinization, inflammation, and colonization of *Propionibacterium acnes* (*P. acnes*) on the pilosebaceous follicles [4]. Among them, inflammations are present in all acne vulgaris lesions—including microcomedones, inflammatory lesions, hyperpigmentation, and scarring [5, 6]. Also, acne vulgaris is associated with diet, occupation, climate, and psychological and lifestyle factors [7, 8]. Thus, more and more researchers are getting interested in preventing acne vulgaris. According to recent dermatologic guidelines, the current treatments for acne vulgaris are divided into conventional pharmacological and nonpharmacological therapies. The former therapies include antibiotics, retinoids, hormonal agents, and benzoyl peroxide while laser and light-based therapies, chemical peels, micro-needling, (micro) dermabrasion, and (mechanical) lesion removal are the latter therapies most commonly applied [9, 10]. Nevertheless, none of these therapies is free of side effect [11]. Furthermore, more investigations are needed to seek alternative and complementary medicine, including medicinal plants.

Bergamot (*Citrus medica* L. var. *sarcodactylis*) has been applied as a medicinal plant just because of its stomachic, antifungal, and bacteriostatic properties [12]. Bergamot, also called finger citron, is one of the species of *Citrus* [13]. The carpels of finger citron split, causing a finger-like fruit shape [14]. The flowers, leaves, and fruits of bergamot can be used as medicine, which play roles in soothing the liver and relieving depression [15]. Bergamot peel is mostly used to distill bergamot essential oil (BEO) while bergamot juice (BJ) is obtained by squeezing the endocarp of the fruits [16]. Evidences indicated that BEO could show some properties like anti-inflammation, immunomodulatory, and wound healing [17]. The flavonoid-rich fraction of BJ could also exhibit anti-inflammatory and antioxidant activities [16]. Sweet orange (*Citrus sinensis* (L.) Osbeck) is another member of the *Citrus* genus fruit which becomes more and more popular in recent years. According to the previous research, sweet orange was widely consumed as fresh fruit and juice, while the peel was also rich in essential oils [18]. Sweet orange juice is one of the natural sources of large amounts of vitamin C, flavonoids, and other bioactive compounds with potential effects on the inflammatory response [19]. Sweet orange essential oil is one of the important natural plant essential oils, with an attractive orange flavor, which was reported to have stress-relief, antifungal, anticarcinogenic, and radical-scavenging properties [20–22]. Moreover, formulations based on orange and sweet basil oils were effective in treating acne vulgaris [23]. Based on the potential physiological activities of bergamot and sweet orange, it is interesting to figure out whether they could show effects on alleviating acne vulgaris. Is it the juice or essential oil that plays roles in improving acne vulgaris? What is the difference between bergamot and sweet orange in ameliorating acne vulgaris?

Therefore, this study was aimed at investigating the effects of different doses of bergamot essential oil, bergamot juice, sweet orange essential oil, and sweet orange juice on acne vulgaris caused by excessive androgen secretion. Since acne vulgaris is associated with elevated levels of androgen, the golden hamster animal model was used in our research, which was frequently used to study acne vulgaris based on the flank sebaceous gland [24–26].

## 2. Materials and Methods

### 2.1. Materials and Reagents

Commercial kits of triglyceride (TG) and enzyme-linked immunosorbent assay (ELISA) commercial kits of caspase-3 were obtained from Nanjing Jiancheng Bioengineering Institute (Nanjing, China), while ELISA kits of testosterone (T), estrogen 2 (E_2_), interleukin-1*α* (IL-1*α*), interleukin-6 (IL-6), tumor necrosis factor-*α* (TNF-*α*), matrix metalloproteinase-2 (MMP-2), and matrix metalloproteinase-9 (MMP-9) were purchased from Keyingmei Biotechnology and Science Inc. (Beijing, China). Other chemical reagents were purchased from Sinopharm Chemical Reagent Co., Ltd. (Beijing, China). Compound pearl acne capsules were produced by Shaanxi Dongtai Pharmaceutical Co., Ltd. (Xianyang, China).

### 2.2. Preparation of Bergamot Juice

After fresh bergamot was cleaned and peeled, peeled bergamot was cut into small pieces of 1 cm × 1 cm × 1 cm to be crushed with the juice extractor. Finally, the bergamot juice was obtained by filtering after placing at 4°C overnight.

### 2.3. Preparation of Bergamot Essential Oil

The peel fractions were diced into small pieces of 8 mm × 8 mm × 1 mm, which were mixed with distilled water at a ratio of 1 : 4 (*m*/*v*). After subjecting small peel fractions to steam distillation for 2 h, the obtained mixtures were dehydrated with anhydrous sodium sulfate. Then, the remaining mixture was strained to remove anhydrous sodium sulfate. Subsequently, the bergamot essential oil was collected after filtration.

### 2.4. Preparation of Sweet Orange Juice

After fresh sweet oranges were cleaned and peeled, peeled oranges were cut into small pieces of 4 cm × 4 cm × 4 cm to be crushed with the juice extractor. Finally, the sweet orange juice was obtained by filtering after placing at 4°C overnight.

### 2.5. Preparation of Sweet Orange Essential Oil

The peel fractions were diced into small pieces of 8 mm × 8 mm × 8 mm, which were mixed with distilled water at a ratio of 1 : 6 (*m*/*v*). After subjecting small peel fractions to steam distillation for 6 h, the obtained mixtures were dehydrated with anhydrous sodium sulfate. Then, the remaining mixture was strained to remove anhydrous sodium sulfate. Subsequently, the sweet orange essential oil was collected after filtration.

### 2.6. Preparation of Solution for Gavage

The prepared essential oil was added to an aqueous solution with 0.2% Tween 80 in a ratio of 1 : 7 (*m*/*v*). Subsequently, the mixtures were ultrasonicated for 30 min to obtain uniform essential oil emulsion. The compound pearl acne capsules were dissolved with distilled water in a ratio of 1 : 6 (*m*/*v*) to prepare an aqueous solution.

### 2.7. Animals and Treatments

Eighty male golden hamsters (120 ± 10 g) were purchased from Beijing Vital River Laboratory Animal Technology Co., Ltd. (Beijing, China) (Certificate SCXK (Beijing) 2012-0001). The hamsters were acclimatized with a daily 12 h light/12 h dark cycle at 23 ± 1°C room temperature and 50 ± 2% relative humidity. After 1 week of adaptation, eighty golden hamsters were randomly divided into 10 groups: model group (MG), positive control group (PG), low-dose group of sweet orange juice (LOJ), high-dose group of sweet orange juice (HOJ), low-dose group of sweet orange essential oil (LOO), high-dose group of sweet orange essential oil (HOO), low-dose group of bergamot juice (LBJ), high-dose group of bergamot juice (HBJ), low-dose group of bergamot essential oil (LBO), and high-dose group of bergamot essential oil (HBO). The entire experiment lasted for four weeks. The MG was given physiological saline by oral route. The PG was administered 0.375 mg/kg BW of compound pearl acne capsules. The LOJ and HOJ were, respectively, orally given 14 and 17.5 mL/kg BW of sweet orange juice. The LOO and HOO received 0.21 and 0.33 mL/kg BW of sweet orange essential oil, respectively. The LBJ and HBJ were, respectively, treated with 14 and 17.5 mL/kg BW of bergamot juice. The LBO and HBO received 0.21 and 0.33 mL/kg BW of sweet bergamot essential oil, respectively. Juice and essential oil that contain a low dose and high dose, respectively, were from fresh fruits in the same weight. The gastric volume of each intervention was 10 mL/kg BW.

At the end of the experiment, the golden hamster was fasted for 12 h (free drinking water) and then deeply comatose with diethyl ether. After taking blood from the eyelids with a capillary tube, hamsters were sacrificed. Blood was stored at 4°C for 4 h and then centrifuged at 4000 g for 10 min in a refrigerated centrifuge. Finally, the supernatant was collected and stored at -80°C for further detection. At the same time, the golden hamsters were dissected and the spleen, testis, and sebaceous gland were taken to weigh them. Part of the sebaceous gland was precooled in liquid nitrogen and then stored in an ultra-low-temperature refrigerator for later detection of related indicators. All experiment procedures in our study involving animals were allowed by the Ethics Committee of the Beijing Key Laboratory of Functional Food from Plant Resources and carried out in accordance with the guidelines for the use and care of laboratory animals of the National Institutes of Health.

### 2.8. Determination of the Growth Rate of Sebaceous Gland Spots

Before the experiment started, the size of sebaceous gland spots on the lateral abdomen of golden hamsters was measured. And the sebaceous gland spots were measured every 7 days. The sebaceous gland spots were calculated as follows:

(1) $$\begin{array}{c} S=\frac{\pi ab}{4}. \end{array}$$

The growth rate of sebaceous gland spots on both sides was calculated as follows:

(2) $$\begin{array}{c} R=\frac{{\text{S}}_{n+1}-{\text{S}}_{n}}{S_{n}}\times 100\%, \end{array}$$

where *a*, *b*, *S*_*n*_, and *S*_*n*+1_ refer to the maximum transverse diameter of the sebaceous gland, the maximum longitudinal diameter of the sebaceous gland, the sebaceous gland area at the *n*-th week, and the sebaceous gland area at the *n* + 1-th week, respectively.

### 2.9. Determination of the Organ Index

After the golden hamsters were weighed and killed, the spleen and testicle weights were measured. The organ index of golden hamsters was calculated as follows:

(3) $$\begin{array}{c} \text{organ index}=\frac{\text{organ weight }\left(\text{mg}\right)}{\text{body weight }\left(\text{g}\right)}. \end{array}$$

### 2.10. TG Analysis

About 0.1 g of sebaceous gland was mixed with physiological saline at a ratio of 1 : 9 (*m*/*v*) and then ground with a homogenizer. The tissue homogenate was prepared in a glass homogenizer and centrifuged at 3000 g for 10 min in a refrigerated centrifuge. The supernatant was taken to detect TG levels following the manufacturer's protocol of the commercial kit.

### 2.11. ELISA Analysis

The serum T, E_2_, IL-1*α*, IL-6, TNF-*α*, MMP-2, and MMP-9 levels and the activity of caspase-3 in sebaceous gland tissue were detected on a SpectraMax M2e enzyme microplate reader (Thermo Fisher Scientific, USA) using the corresponding ELISA kits following the manufacturer's instructions.

### 2.12. Statistical Analysis

All diagrams were generated using GraphPad Prism 8.0 (GraphPad Software, San Diego, CA, USA). Statistical data were analyzed by one-way analysis of variance (ANOVA) followed by Tukey's test using SPSS 25.0 software (IBM Corporation, Armonk, NY, USA). Results were expressed as mean ± standard deviation (SD) with significance accepted at *P* < 0.05.

## 3. Results

### 3.1. Effect of Different Treatments on the Growth Rate of Sebaceous Gland Spots in the Golden Hamster

**Figure 1:**
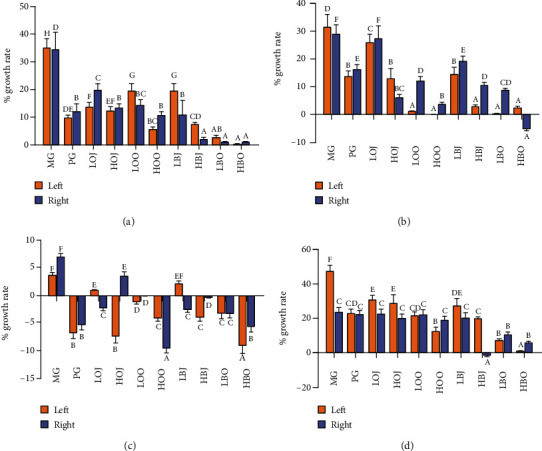
Effect of different treatments on the growth rate of sebaceous gland spots: (a) the growth rate of sebaceous gland spots in the first week, (b) the growth rate of sebaceous gland spots in the second week, (c) the growth rate of sebaceous gland spots in the third week, and (d) the growth rate of sebaceous gland spots in the fourth week. Values are expressed as the mean ± SD (*n* = 8). Data with different letters indicate significant differences (*P* < 0.05).

As shown in Figure 1(a), all groups exhibited better inhibitory effect than MG on the growth rate of sebaceous gland spots (*P* < 0.05). The growth rate of the sebaceous gland spots in bergamot essential oil groups was remarkably decreased compared with the corresponding dose of juice groups. HBO, LBO, and HOO could significantly inhibit the growth rate of the left-side sebaceous gland spots of hamsters compared with the PG group (*P* < 0.05). The HBO, LBO, and HBJ groups significantly decreased the growth rate of the right-side sebaceous gland spots in golden hamsters to 2.21%, 1.13%, and 1.09%, respectively (*P* < 0.05). The growth rate of the sebaceous gland spots in bergamot essential oil groups was clearly decreased in contrast with the corresponding dose of sweet orange groups (*P* < 0.05). In Figure 1(b), each intervention group clearly reduced the growth rate of the left-side sebaceous gland spots compared with MG (*P* < 0.05). Only the groups of bergamots, LOO, HOO, and HOJ, could significantly inhibit the growth rate of right-side sebaceous gland spots in comparison with MG (*P* < 0.05). The growth rate of sebaceous gland spots in essential oil groups was lower than that in the corresponding dose of juice groups. According to Figure 1(c), the growth rate of the right-side sebaceous gland of each intervention group was significantly different from that of the MG (*P* < 0.05), while the growth rate of the left-side sebaceous gland of LBO, HBO, HBJ, and sweet orange groups was significantly lower than that of the MG (*P* < 0.05). The growth rate of the sebaceous gland spots in sweet orange essential oil groups was obviously decreased in contrast with the corresponding dose of juice groups (*P* < 0.05). In Figure 1(d), compared with the MG, each intervention group significantly reduced the growth rate of the left-side sebaceous gland spots of the golden hamster (*P* < 0.05). In contrast, only the groups of LBO, HBO, and HBJ could significantly decrease the growth rate of the right-side sebaceous gland spots in contrast with MG (*P* < 0.05). Treatment with sweet orange essential oil could significantly decrease the growth rate of the sebaceous gland spots compared with the corresponding dose of juice groups (*P* < 0.05). Both of bergamot and sweet orange groups exhibited the effect of decreasing the growth rate of sebaceous gland spots with a dose-effect relationship.

### 3.2. Effect of Different Treatments on the Organ Index in the Golden Hamster

As listed in Table 1, there was no significant difference in the testicular index and spleen index between the intervention groups and MG. These results indicated that the intervention substances did not have adverse effects on the testicular and spleen indexes in golden hamsters.

### 3.3. Effect of Different Treatments on the Level of Serum T and E_2_ in the Golden Hamster

**Figure 2:**
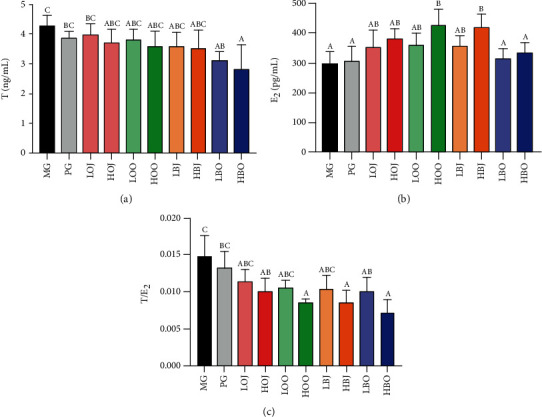
Effect of different treatments on serum sex hormone levels: (a) serum T level, (b) serum E_2_ level, and (c) the ratio of T/E_2_. Values are expressed as the mean ± SD (*n* = 8). Significance was defined as *P* < 0.05.

The changes in T level of golden hamsters are shown in Figure 2(a). Compared with the MG, the content of T in each intervention group showed a certain degree of reduction. However, only the bergamot essential oil groups significantly reduced the T level in comparison with MG (*P* < 0.05). Besides, all intervention groups increased the E_2_ content compared with MG (shown in Figure 2(b)). But only HOO and HBJ could remarkably increase the E_2_ content compared with MG (*P* < 0.05). These results did not show a statistical difference between the sweet orange groups. In Figure 2(c), data showed that each intervention group lowered the ratio of T/E_2_ in comparison with MG. Among them, the ratio of T/E_2_ in high-dose intervention groups was significantly decreased in comparison with that in MG (*P* < 0.05). The ratio of T/E_2_ in essential oil groups was lower than that in the corresponding dose of juice groups. Both of bergamot and sweet orange groups had shown a dosage-effect relationship.

### 3.4. Effect of Different Treatments on the Level of TG of Sebaceous Gland Tissue in the Golden Hamster

**Figure 3:**
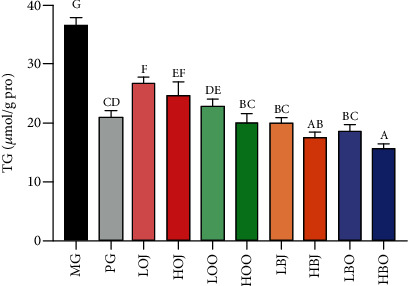
Effect of different treatments on the level of TG. Values are expressed as the mean ± SD (*n* = 8). Data with different letters indicate significant differences (*P* < 0.05).

The results of TG level are shown in Figure 3. Compared with the MG, each intervention group significantly reduced the content of TG in the sebaceous gland of golden hamsters (*P* < 0.05). As can be seen from Figure 3, the TG level in sweet orange essential oil groups was obviously decreased in contrast with the corresponding dose of juice groups (*P* < 0.05). The content of TG in bergamot essential oil groups was lower than that in the corresponding dose of juice groups. Both of bergamot and sweet orange groups exhibited effects on attenuating TG level with a dose-effect relationship.

### 3.5. Effect of Different Treatments on Inflammatory Factors in the Golden Hamster

**Figure 4:**
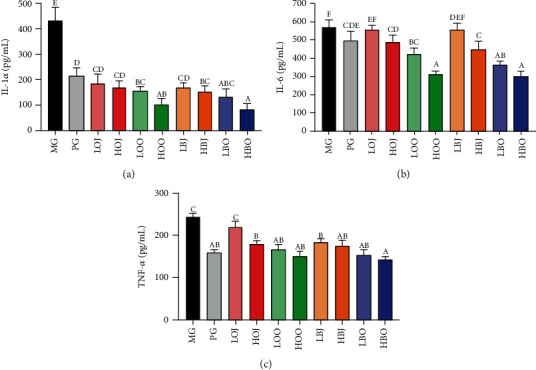
Effect of different treatments on the level of serum inflammatory factors in the golden hamster: (a) IL-1*α* level, (b) IL-6 level, and (c) TNF-*α* level. Values are expressed as the mean ± SD (*n* = 8). Data with different letters indicate significant differences (*P* < 0.05).

In this part, we studied the effect of different treatments in the golden hamster on the level of the serum inflammatory factor (shown in Figure 4). In Figure 4(a), data showed that each intervention group clearly lowered the serum IL-1*α* content in comparison with MG (*P* < 0.05). The HBO even lowered the IL-1*α* level to about one-fifth of the MG (83 pg/mL and 433 pg/mL, respectively). The IL-1*α* level in HOO was obviously reduced relative to that in HOJ. Meanwhile, bergamot groups showed similar results. As shown in Figure 4(b), the content of IL-6 was clearly decreased in HBO, LBO, and HOO groups in contrast with MG (*P* < 0.05). Groups with essential oil could significantly decrease IL-6 level compared with the corresponding dose of juice groups (*P* < 0.05). As shown in Figure 4(c), in addition to LOJ, all other intervention groups significantly reduced TNF-*α* level (*P* < 0.05). Treatment with low-dose sweet essential oil could significantly reduce TNF-*α* level relative to that with LOJ (*P* < 0.05). The TNF-*α* level in essential oil groups was lower than that in the corresponding dose of juice groups. Both of bergamot and sweet orange groups had anti-inflammation effect by a quantity-effect relationship.

### 3.6. Effect of Different Treatments on the Level of Serum MMPs in the Golden Hamster

**Figure 5:**
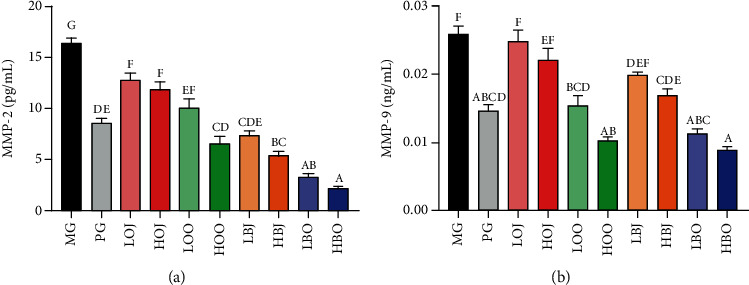
Effect of different treatments on the level of serum MMP-2 and MMP-9 in the golden hamster: (a) MMP-2 level and (b) MMP-9 level. Values are expressed as the mean ± SD (*n* = 8). Significance was defined as *P* < 0.05.

We found that each intervention group significantly reduced the MMP-2 content in comparison with the MG (*P* < 0.05). As shown in Figure 5(a), the content of MMP-2 in HOO groups was obviously decreased in contrast with that in HOJ (*P* < 0.05). Groups with bergamot essential oil could significantly decrease MMP-2 level compared with the corresponding dose of juice groups (*P* < 0.05). In Figure 5(b), the content of MMP-9 in the essential oil groups could be reduced to about half of that in the MG. Groups with essential oil could significantly reduce MMP-9 level in contrast with the corresponding dose of juice groups (*P* < 0.05). Both of bergamot and sweet orange groups had attenuating effects on MMP level with a dose-effect relationship.

### 3.7. Effect of Different Treatments on the Activity of Caspase-3 of Sebaceous Gland Tissue in the Golden Hamster

**Figure 6:**
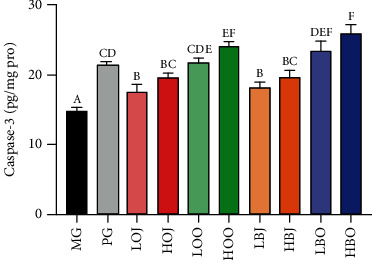
Effect of different treatments in the golden hamster on the activity of caspase-3. Values are expressed as the mean ± SD (*n* = 8). Significance was defined as *P* < 0.05.

Caspase-3 levels were determined to assess the effect of different treatments on apoptosis of sebaceous gland cells. As shown in Figure 6, we found that each intervention group significantly increased the relative activity of caspase-3 in comparison with the MG (*P* < 0.05). Groups with essential oil could significantly increase the relative activity in comparison with the corresponding dose of juice groups (*P* < 0.05). Bergamot and sweet orange groups could increase the relative activity of caspase-3 with a dosage-effect relationship.

## 4. Discussion

Golden hamsters could be used as a model of acne vulgaris caused by the excessive secretion of androgen, which appeared in two sebaceous glands in the lateral abdomen [27]. Therefore, determining the area and thickness of the sebaceous gland can reflect the severity of acne vulgaris in golden hamsters, to a certain extent. It was reported that *Lactobacillus*-fermented *C. obtuse* significantly decreased sebum secretion and size of the sebaceous gland compared with baseline (0.24 ± 0.09 mm^2^ vs. 0.38 ± 0.11 mm^2^, respectively) [28]. In our study, the size of the sebaceous gland was not reduced with the treatment with bergamot and sweet orange, and the growth rate of sebaceous gland spots decreased instead. It was reported that activating the expression of apoptotic proteins through a series of reactions could ultimately lead to a reduction in the area or thickness of the sebaceous gland [29]. Caspase-3 plays an executing role in the process of apoptosis, and it is also the most important downstream protease of the apoptotic effect and execution process [30]. Inhibition of caspase-3 was reported to attenuate sebaceous gland cell proliferation and organ size [31]. In this study, the growth rate of sebaceous gland spots and caspase-3 activity in golden hamsters with different doses of intervention substances were analyzed. It was found that after 4 weeks of intervention, the growth rate of sebaceous gland spots in each intervention group was significantly decreased compared with that in MG, and caspase-3 activity was significantly increased in comparison with that in MG (Figures 1 and 6). Moreover, the effect of each intervention substance was positively correlated with the dose, and the bergamot essential oil had the best improvement effect. Therefore, it is suggested that bergamot essential oil, bergamot juice, sweet orange essential oil, and sweet orange juice may alleviate acne vulgaris by promoting the apoptosis of sebaceous gland cells in the golden hamster through enhancing the activity of caspase-3.

The secretion of androgen plays an important role in the occurrence and development of acne lesions [11]. Excessive androgen secretion or imbalance of androgen and estrogen levels can lead to sebaceous gland hyperplasia, excessive sebum secretion, and abnormal keratinization of the hair follicle sebaceous gland, resulting in the development and continuous occurrence of acne vulgaris [32]. High-dose estrogen exerted a negative feedback on the gonadal axis, which could result in the reduction of sebum formation [33]. Evidence showed that higher content of free T with lower level of E_2_ was significantly in favor of severe acne vulgaris [34]. Thus, balancing the ratio between androgens and estrogens could help alleviate or treat acne vulgaris. In our study, the bergamot essential oil significantly reduced the serum T content in golden hamsters, while HOO and HOJ groups markedly increased the serum E_2_ level (Figures 2(a) and 2(b)). This indicated that sweet orange essential oil and bergamot essential oil could improve acne lesions via directly reducing the androgen level or decreasing the androgen/estrogen ratio (Figure 2(c)).

Sebum is synthesized and secreted by sebaceous gland cells, consisting of TG, nonesterified fatty acid, wax ester, squalene, and cholesterol ester [35]. Since sebum contains approximately 30% TG, the amount of sebum synthesis and secretion was reflected in the study by measuring the TG content in the sebaceous gland tissue of golden hamsters. The study found that bergamot essential oil, bergamot juice, sweet orange essential oil, and sweet orange juice can significantly reduce the TG content of golden hamsters, indicating that each intervention substance can improve acne vulgaris via restraining TG level to a certain extent (Figure 3). Besides, the bergamot essential oil, bergamot juice, sweet orange juice, and sweet orange essential oil exhibited a dose-dependent effect on inhibiting TG accumulation.

Inflammation is an initial host immune reaction which is mediated by inflammatory factors [36]. The induction of inflammation mediators is associated with *P. acnes* [37]. *P. acnes* and associated cellular membrane lipopolysaccharides could induce the expression of IL-8, TNF, and IL-1*α* in cultured sebocytes [38]. The underlying mechanisms are that sebocytes can be recognized and activated by *P. acnes*, which further trigger the release of inflammatory cytokines [39]. TNF-*α* is an extensively studied cytokine associated with many inflammatory diseases [40]. Evidences indicated that IL-1*α* is one of the main inflammatory factors, which could induce the proliferation of keratinocytes and sebaceous gland cells and eventually promote the formation of acne vulgaris [41]. Moreover, increased IL-1 contents could further promote the expression of MMPs and lipid synthesis [42, 43]. MMPs have been suggested to be involved in acne vulgaris, and different levels of MMP expression could contribute to the development of different types of acne lesions [44–46]. Keratinocytes are an important source of MMPs in acne vulgaris, and *P. acnes* can induce the expression of several kinds of MMPs, including MMP-2 and MMP-9 [47]. Kim et al. reported that *Citrus obovoides* and *Citrus natsudaidai* essential oils could reduce *P. acnes*-induced secretion of IL-8 and TNF-*α* [48]. In one study, a *Lactobacillus*-fermented *Chamaecyparis obtusa* leaf extract was reported to have a great effect on inflammatory markers, such as IL-8, IL-1, and NF-*κ*B [28]. Also, previous studies reported that essential oil and juice could alleviate acne vulgaris based on their antibacterial effect [49, 50]. In our study, results showed that bergamot essential oil, bergamot juice, sweet orange essential oil, and sweet orange juice could reduce the levels of IL-6, TNF-*α*, MMP-2, MMP-9, and especially IL-1*α*, indicating that bergamot essential oil, bergamot juice, sweet orange essential oil, and sweet orange juice could improve acne vulgaris through alleviating inflammatory response and suppressing *P. acnes* in golden hamsters (Figures 4 and 5).

## 5. Conclusions

In summary, bergamot essential oil, bergamot juice, sweet orange essential oil, and sweet orange juice could improve acne vulgaris caused by excessive secretion of androgen via reducing the growth rate of the sebaceous gland, inhibiting TG accumulation and inflammatory cytokines release in the sebaceous gland, promoting apoptosis in the sebaceous gland, and decreasing the ratio of T/E_2_. In general, bergamot essential oil and sweet orange essential oil may have better effects on alleviating acne vulgaris than the corresponding juice. Among them, the levels of IL-1*α* in the sebaceous gland could be best reduced in both bergamot and sweet orange. This implies that bergamot and sweet orange may improve acne lesions via alleviating inflammatory response and suppressing *P. acnes*, but further studies of the mechanism are needed.

## Figures and Tables

**Table 1:** Effect of different treatments on the organ index.

Group	Testicular index (mg/g)	Spleen index (mg/g)
MG	34.41 ± 2.09^a^	0.72 ± 0.05^a^
PG	32.54 ± 1.33^a^	0.78 ± 0.09^a^
LOJ	31.14 ± 2.63^a^	0.71 ± 0.17^a^
HOJ	31.87 ± 3.08^a^	0.78 ± 0.08^a^
LOO	33.09 ± 2.48^a^	0.71 ± 0.07^a^
HOO	34.16 ± 2.39^a^	0.74 ± 0.08^a^
LBJ	32.07 ± 2.20^a^	0.70 ± 0.08^a^
HBJ	33.57 ± 3.25^a^	0.71 ± 0.08^a^
LBO	33.66 ± 2.80^a^	0.71 ± 0.01^a^
HBO	34.14 ± 2.60^a^	0.70 ± 0.11^a^

Values are expressed as the mean ± SD (*n* = 8). Data with different letters on the same line indicate significant differences (*P* < 0.05).

## Data Availability

The data used to support the findings of this study are available from the corresponding author upon request.
